# Characterization of bacterial communities associated with blood-fed and starved tropical bed bugs, *Cimex hemipterus* (F.) (Hemiptera): a high throughput metabarcoding analysis

**DOI:** 10.1038/s41598-021-87946-w

**Published:** 2021-04-19

**Authors:** Li Lim, Abdul Hafiz Ab Majid

**Affiliations:** Household and Structural Urban Entomology Laboratory, Vector Control Research Unit, School of Biological Sciences, Universiti Sains Malaysia, 11800 Minden, Penang Malaysia

**Keywords:** Zoology, Entomology, Microbial communities

## Abstract

With the development of new metagenomic techniques, the microbial community structure of common bed bugs, *Cimex lectularius*, is well-studied, while information regarding the constituents of the bacterial communities associated with tropical bed bugs, *Cimex hemipterus*, is lacking. In this study, the bacteria communities in the blood-fed and starved tropical bed bugs were analysed and characterized by amplifying the v3-v4 hypervariable region of the 16S rRNA gene region, followed by MiSeq Illumina sequencing. Across all samples, Proteobacteria made up more than 99% of the microbial community. An alpha-proteobacterium *Wolbachia* and gamma-proteobacterium, including *Dickeya chrysanthemi* and *Pseudomonas*, were the dominant OTUs at the genus level. Although the dominant OTUs of bacterial communities of blood-fed and starved bed bugs were the same, bacterial genera present in lower numbers were varied. The bacteria load in starved bed bugs was also higher than blood-fed bed bugs.

## Introduction

*Cimex hemipterus* Fabricus (Hemiptera), also known as tropical bed bugs, is an obligate blood-feeding insect throughout their entire developmental cycle, has made a recent resurgence probably due to increased worldwide travel, climate change, and resistance to insecticides^1–3^. Distribution of tropical bed bugs is inclined to tropical regions, and infestation usually occurs in human dwellings such as dormitories and hotels^1,2^. Bed bugs are a nuisance pest to humans as people that are bitten by this insect may experience allergic reactions, iron deficiency, and secondary bacterial infection from bite sores^4,5^.

Eradication of bed bugs from an infested area is challenging not only because they are resistant to insecticides but also because they can crawl and hide into tight crevices, making them difficult to discover^4,6^. Moreover, bed bugs could survive for many months without feeding, causing more problems for the residents^4^. The various mechanisms, including metabolic, behavioural, and physiological of bed bugs, could have contributed to maintaining wellness^4,5^. Nevertheless, the role played by microbiota may be as well crucial in bed bugs’ survival.

Insects are commonly possessing symbiotic microorganisms that essential in host’s growth, development, nutrient supplement, protection, insecticides resistance, and reproduction^5,7^. For example, *Buchnera*, an obligate symbiont of aphids, is essential in maintaining their host^8^. Due to a restricted diet, bed bugs are not unusual to maintain symbiotic microorganisms that played roles in their survival^1,9^. *Wolbachia* is indeed a confirmed endosymbiont in common bed bugs, *Cimex lectularius*, which is essential in reproduction and provides the B vitamins necessary for growth^10^.

Metagenomics is the study to acquire a general view of the microbial community composition in certain eco-environments via sequence the whole genetic material of the microbial community^11^. With the development of next-generation sequencing (NGS) and bioinformatics, metagenomics study becomes more convenient, efficient, cost-effective and overcome the problem of which most of the microorganism cannot be isolated and maintained in pure culture^12,13^. Many metagenomic studies of arthropods including mosquito^14^, tick^15^, assassin bugs^16^, tsetse flies^17^ as well as common bed bugs^18^ had used the sequencing of 16S rRNA gene to characterize the microbiota. However, the microbiome of tropical bed bugs, *C. hemipterus*, remains mostly unexplored, and the application of high-throughput sequencing in the characterization of bacteria community in tropical bed bugs is not available.

In order to understand how endosymbiotic bacteria in contributing to their hosts’ wellness, characterization of the bacterial community in *C. hemipterus* is a preliminary step before further investigations. As the limitation of blood meals or starvation faced by bed bugs may affect the structure and relative abundance of bacteria community that reside within their host due to nutrients depletion^19^, characterizing the bacterial composition of bed bugs during starvation may as well imperative, given that these bacteria may play an essential role in their host survival. Thus, both bacterial compositions in blood-fed and starved *C. hemipterus* were analysed and characterized using 16S rRNA PCR amplification followed by Miseq Illumina sequencing.

## Materials and methods

### Bed bugs

*Cimex hemipterus* that used in this study was originated from specimens collected in Kuala Lumpur International Airport (KLIA) back in 2014 and maintained since that time at the Household and Structural Urban Entomology Laboratory, Vector Control Research Unit, School of Biological Sciences, Universiti Sains Malaysia^3^. The bed bugs colony was maintained in plastic containers (200 ml) covered with cloth mesh at 23 ± 1 °C and fed on the volunteer arm directly once every two weeks. The feeding of bed bugs on human blood followed the protocol with code, USM/JEPeM/19120868, approved by the Human Research Ethics Committee USM (HREC).

### DNA extraction and sequencing

Male, adult stage bed bugs were used in this experiment. Two tested treatments: bed bugs under blood-fed state (bed bugs were euthanized and preserved after feeding) and bed bugs under a starved state (bed bugs were euthanized and preserved 45 days after feeding). There were three biological replications for each treatment with only one sample (one individual male adult tropical bed bug) in each replication. The samples were preserved in absolute ethanol in the freezer under  − 20 °C. Before DNA isolation, each bed bug preserved in absolute ethanol was surface sterilized with 70% ethanol for 15 min and then washed with sterile distilled water. The bed bug sample was then placed in a new 1.5 ml microcentrifuge tube and homogenized with a micro pestle.

Microbial DNA was extracted from the homogenized bed bug sample using the HiYield Genomic DNA isolation kit (Real Biotech Corporation, Taiwan) following the manufacturer's standard protocol. DNA concentration and quality were assessed using Quawell Micro Volume Spectrophotometer (Labgene Scientific, Switzerland). DNA extracts' integrity and purity were assessed through visualized on 1% (w/v) agarose gels. 16S v3-v4 amplicon metagenomics paired-end sequencing was conducted on the Illumina MiSeq platform following the standard protocol. The raw sequence data is accessible at the NCBI Sequence Read Archive (SRA) database under the bioproject number PRJNA600667.

### Bioinformatics analysis

The quality of generated sequences or raw fastq files was assessed using QIIME (version 1.9.0)^20^. Trimmomatic^21^ was subsequently used to trim the sequences at any site receiving an average quality score less than 20 and discard those shorter than 50 bp. Sequences that overlap longer than ten bp were assembled according to their overlap sequence using FLASH^22^. Operational taxonomic units (OTUs) were clustered with 97% similarity cut-off using UPARSE^23^ while UCHIME^24^ was used to identified and removed chimeric sequences. The taxonomy of each 16S rRNA gene sequence was analysed using RDP Classifier^25^ against the Silva 16S rRNA database^26^. The taxon identity from kingdom to species level was then assigned to the representative OTU sequences. 16S rRNA gene sequences were also aligned to the Parallel-META 3^27^ reference database for OTU picking and taxonomical annotation. The rarefaction curve was plotted using R software^28^ to determine the sequencing depth sufficiency. Heatmap showed that the percentage of microorganisms in a matrix was also derived using R software^28^.

Shannon, Simpson (Simpson's Index of Diversity), and CHAO1 estimators were used to measure the alpha-diversity of blood-fed and starved bed bugs using the Parallel-Meta 3 pipeline. The results were presented through boxplots by R software. Significant differences (*P* < 0.05) in diversity indexes were determined using the pairwise Wilcoxon test. The observed number of OTUs also used as an indicator of species richness.

Beta-diversity differences were calculated based on the Bray–Curtis, Weighted-, and Unweighted-UniFrac distance matrices. Principal coordinates analysis (PCoA) plots were generated based on Weighted- and Unweighted-UniFrac distance matrices using R software^28^. Mantel test with 999 permutations was used to compare the dissimilarity of distance matrices between blood-fed and starved bed bugs and determine the R statistics, and the *P*-value with *P*-values less than 0.05 were considered statistically significant.

The number of sequences at the genus level between blood-fed and starved bed bugs was also compared and screened the significantly different bacterial taxa using a paired sample T-test. Taxa that present in both blood-fed and starved bed bugs were selected for this analysis.

### Ethical approval

Household and Structural Urban Entomology laboratory research mainly focus on urban pests, including household ants, termites, cockroaches, and bed bugs. However, to rear and breed the bed bugs sufficient for different experiments, including physiology, molecular, and insecticide resistance studies, human blood-feeding for bed bugs is unavoidable since studies have shown bed bugs with human blood-feeding have a higher percentage in profuse breeding.

The participant that takes part in bed bugs blood feeding is on his freewill. The participant is well-educated and well-aware of the possible risks regarding direct blood-feeding. We then obtained informed consent from the participant. No allergy or any medical issues have arisen of bed bugs being direct feed on the volunteer's arm.

Our research protocol has been reviewed and approved for implementation by The Human Research Ethics Committee of USM or Jawatankuasa Etika Penyelidikan Manusia Universiti Sains Malaysia (JEPeM) with assigned study protocol code USM/JEPeM/19120868. JEPeM-USM complies with the Declaration of Helsinki, International Conference on Harmonization (ICH) Guidelines, Good Clinical Practice (GCP) Standards, Council for International Organizations of Medical Sciences (CIOMS) guidelines, World Health Organization (WHO) Standards and Operational Guidance for Ethics Review of Health-Related Research and Surveying and Evaluating Ethical Review Practices, EC/IRB Standard Operating Procedures (SOPs), and Local Regulations and Standards in Ethical Review. Statement regarding the participant's informed consent and JEPeM approval has been added in the manuscript's method section.

## Results

### Data summary

The general characteristics of the sequencing datasets were shown in Table 1. For bed bugs samples in the blood-fed state, the dataset consisted of 32,816, 43,211, and 32,171 sequences with an average read lengths of 413.50, 411.61, and 413.13 from individuals BB1, BB2, and BB3, respectively. A total of 138,298, 109,834, and 125,804 reads were generated with average read lengths of 409.79, 409.41, and 408.98 from starved bed bugs samples BB1, BB2, and BB3, respectively. The higher number of OTUs in starved bed bugs indicated higher species richness (Table 1).

**Table 1:** General characteristics of sequencing data for each bed bug sample.

State	Sample	Number of reads	Average length of reads	Number of OTUs
Blood fed	BB1	32,816	413.50	29
	BB2	43,211	411.61	18
	BB3	32,171	413.13	28
Starved	BB1	138,298	409.79	154
	BB2	109,834	409.41	52
	BB3	125,804	408.98	34

*BB1-BB3 were replicates.

**Figure 1:**
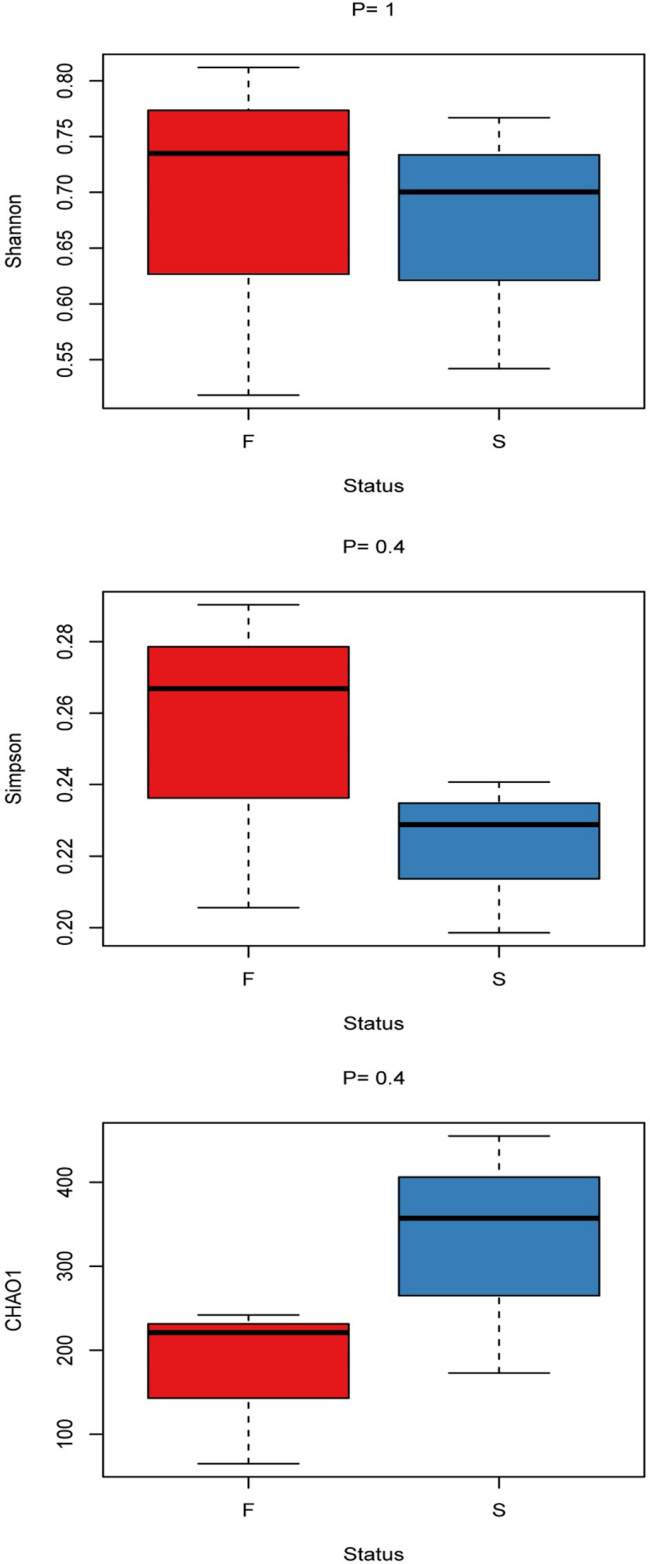
Alpha-diversity boxplot of blood-fed (F, red) and starved (S, blue) bed bugs.

Both Shannon and Simpson indices indicate higher species richness in blood-fed bed bugs. However, the Chao 1 indices estimated greater species richness for starved bed bugs. As a whole, although there is variability in the richness of the bacterial assemblages between blood-fed and starved bed bugs, comparisons of the sequences using several alpha diversity metrics including Shannon, Simpson, and CHAO1, demonstrated in the boxplot revealed no significant differences (Shannon, *p* = 1; Simpson, *p* = 0.4; CHAO1, *p* = 0.4) between blood-fed and starved bed bugs of which supported by pairwise Wilcoxon test (Fig. 1).

**Figure 2:**
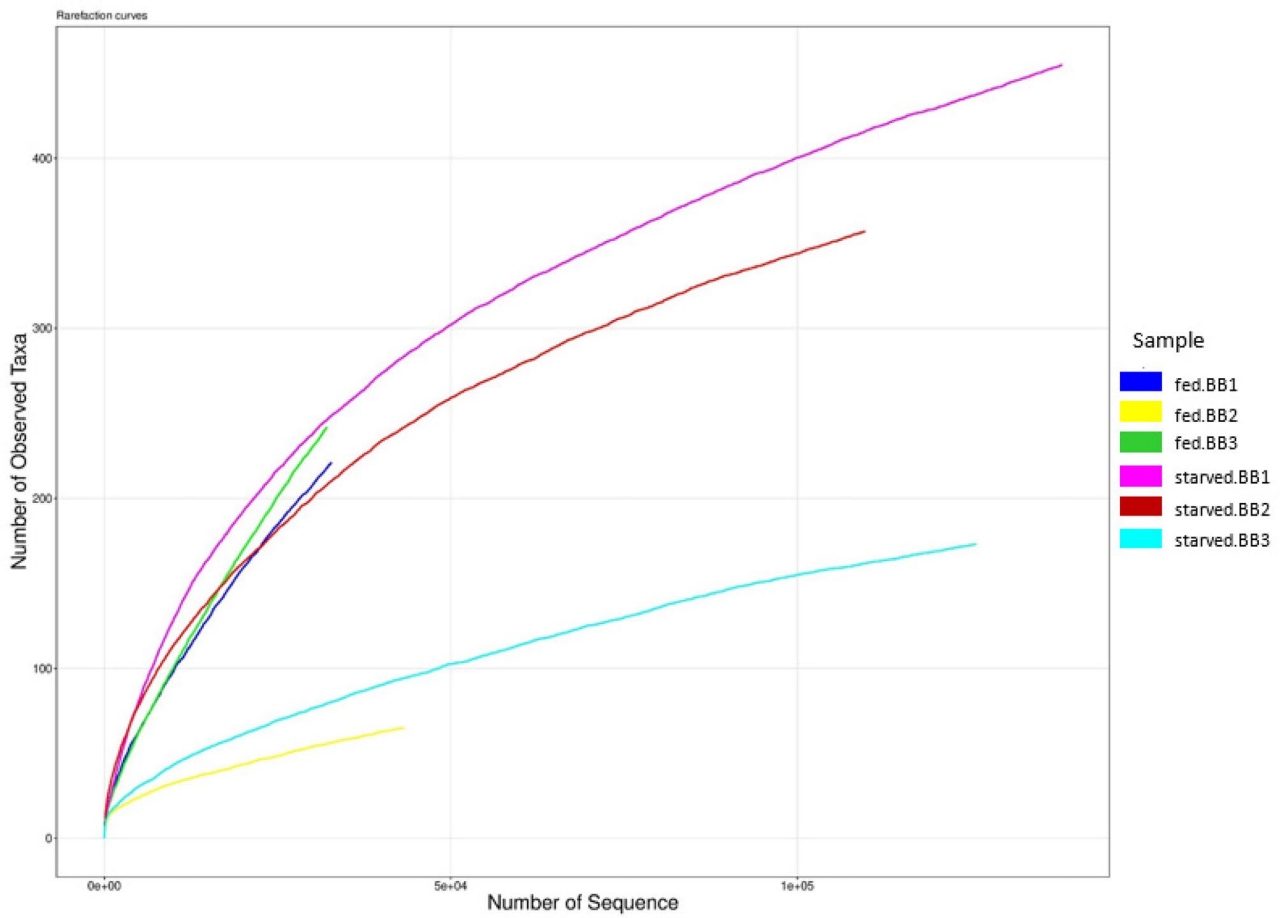
Rarefaction curves of the three samples, fed.BB1 (blue line), fed.BB2 (yellow line) and fed.BB3 (green line) from blood-fed bed bugs and three samples, BB1 (purple line), BB2 (red line) and BB3 (turquoise line) from starved bed bugs.

Nevertheless, the rarefaction curves generated by plotting the number of observed taxa against the number of sequences derived through Parallel-META 3 showed starved bed bugs generally had steeper and higher curves than blood-fed bed bugs indicated higher taxa richness (Fig. 2). Except for curves of samples fed.BB2 and fed.BB3, others in Fig. 2 showed plateau or approached saturation, indicating that adequate sequencing depth was achieved^13^. The curves that have not reached a plateau indicating more undetected OTUs may exist in these samples, which could probably affect the Shannon and Simpson indices.

**Figure 3:**
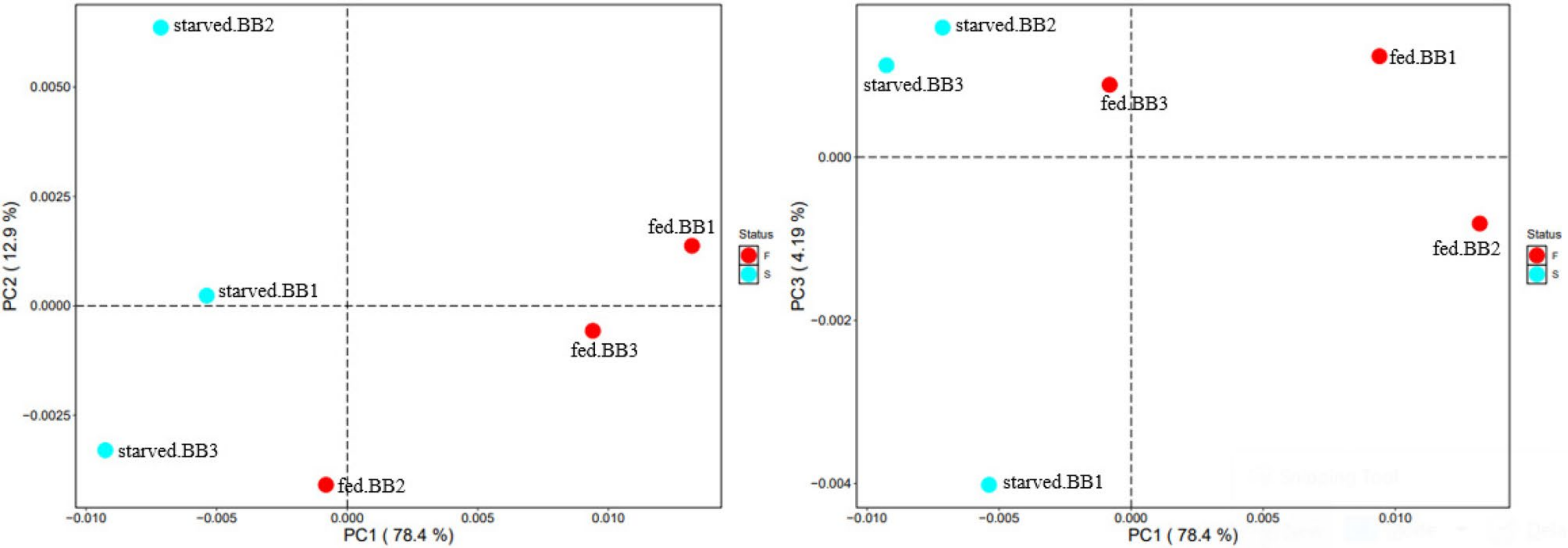
Principal coordinates analysis (PCoA) using Weighted-UniFrac distances of bacterial communities of blood-fed (red points) and starved (turquoise points) bed bugs.

The principal coordinates analysis (PCoA) plots shown beta-diversity differences between each blood-fed and starved bed bug sample based on Weighted-UniFrac distances matrix (Fig. 3). The first three PCoA axes with PC1-78.4%, PC2-12.9%, and PC3-4.19% based on Weighted-UniFrac explain the bacterial community variance among blood-fed and starved bed bugs. Points that are ordinated closer together in the plots indicate more miniature dissimilarity community composition than those ordinated farther apart. According to tropical bed bugs' physiological status, clustering was observed (Fig. 3), indicating dissimilarity and difference in the bacterial community between samples of blood-fed and starved bed bugs. Nevertheless, the Mantel test reveal that the dissimilarity of bacterial community had no significant difference between blood-fed and starved bed bugs based on Weighted-Unifrac (r =  − 0.100; *p* = 0.156) and Unweighted-UniFrac (r =  − 0.579; *p* = 0.702) measures. This pattern also held with Bray–Curtis community dissimilarity matrices (r =  − 0.224; *p* = 1.000).

The rather far apart of each point also presented inter-individual variability within both treatment groups (Fig. 3). However, the variability between individuals also has no significant difference (data not shown).

### Bacterial community

**Figure 4:**
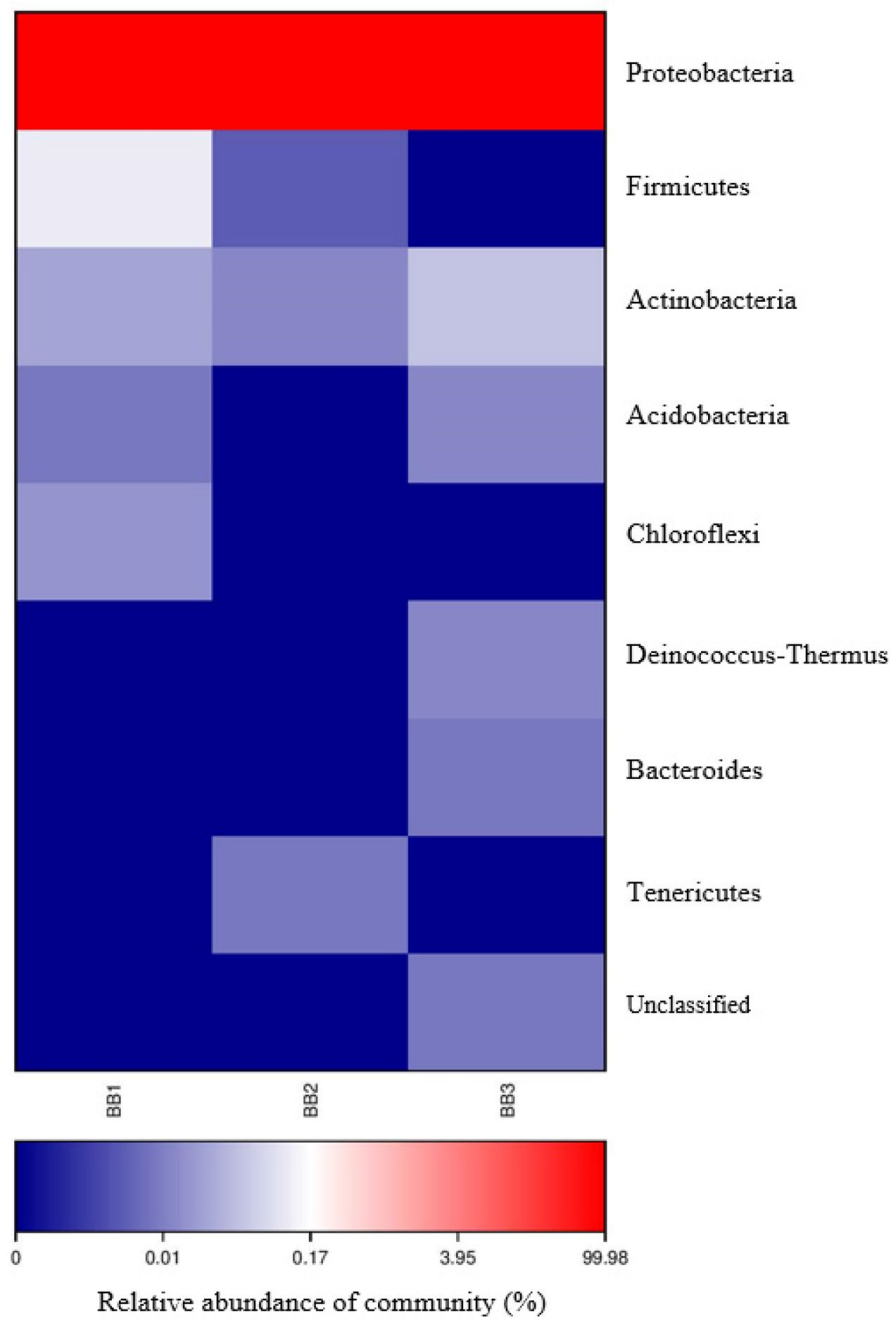
Heatmap analysis of three samples, BB1, BB2 and BB3 from blood-fed bed bugs.

**Figure 5:**
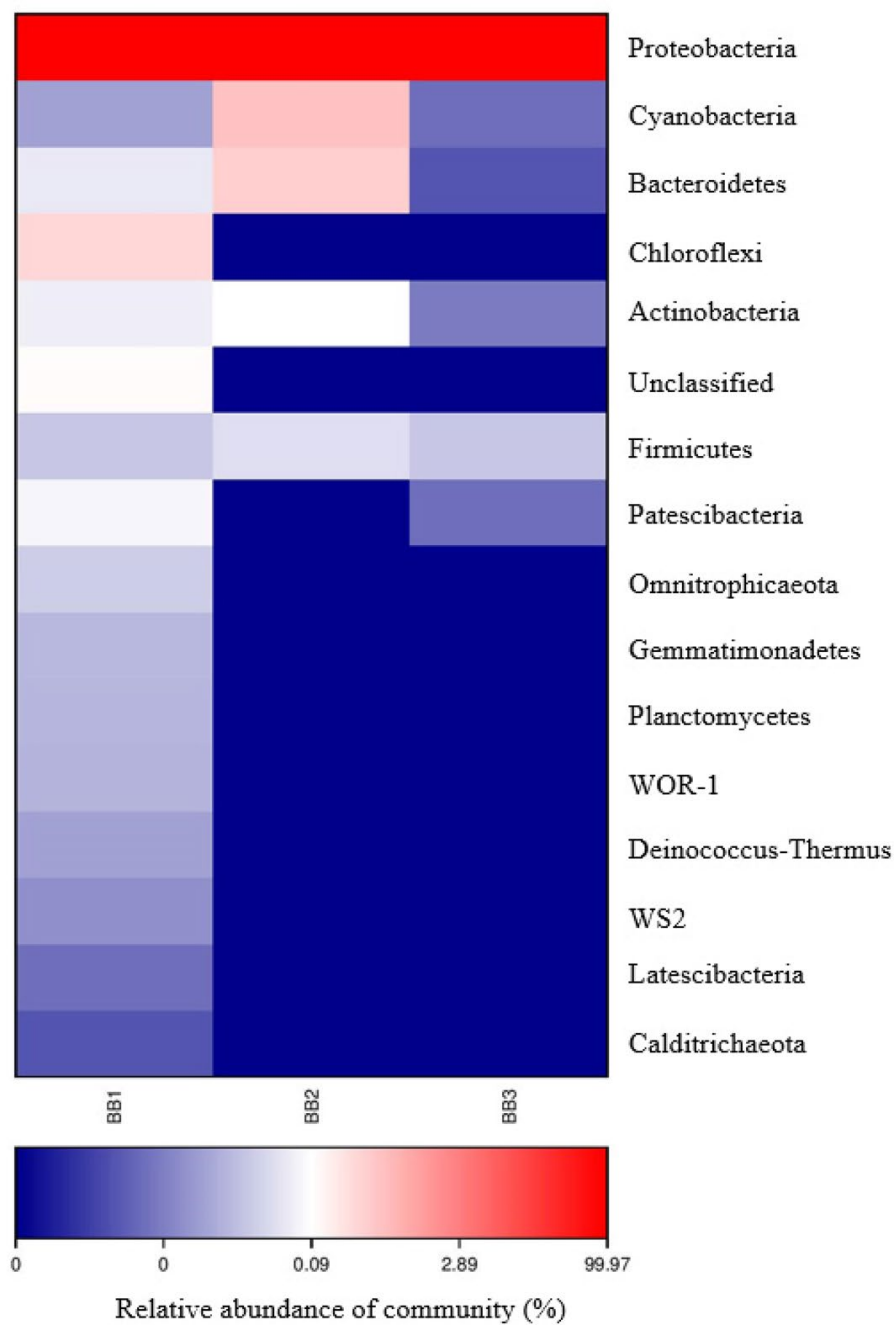
Heatmap analysis of three samples, BB1, BB2 and BB3 from starved bed bugs.

Heatmap revealed that Proteobacteria was the dominant phylum with relative abundances more than 99% across all samples from both blood-fed (Fig. 4) and starved (Fig. 5) bed bugs. For blood-fed bed bugs, the remaining OTUs (less than 1%) were classified into the phyla Firmicutes, Actinobacteria, Acidobacteria, Chloroflexi, Deinococcus-Thermus, Bacteroidetes, Tenericutes or were unassigned at the phylum level (Fig. 4).

The assigned phylum for remaining OTUs of starved bed bugs was slightly different from blood-fed bed bugs and more diversified. After Proteobacteria, Cyanobacteria was the second-highest abundance in starved bed bugs, followed by Bacteroidetes, Chloroflexi, Actinobacteria, Unclassified phylum, Firmicutes, Patescibacteria, Omnitrophicaeota, Gemmatimonadetes, Planctomycetes, WOR-1, Deinococcus-Thermus, WS2, Latescibacteria, and Calditrichaeota (Fig. 5).

The bacterial genera shared between the three samples, BB1, BB2, and BB3 of blood-fed and starved bed bugs was shown in Table 2. The relative abundance of bacterial taxa identified refers to the proportion of reads for each taxon relative to the total number of reads for all taxa. Alpha-proteobacteria and gamma-proteobacteria were dominant at the class level. There were seven bacterial genera, including *Wolbachia*, *Dickeya chrysanthemi*, *Pseudomonas*, *Enhydrobacter*, *Paracoccus*, *Methylobacterium*, and *Curvibacter* were present in both blood-fed and staved bed bugs. All blood-fed samples shared four bacterial genera: *Shimwellia*, *Skermanella*, *Pelomonas*, and *Mesorhizobium*, which exhibited a low relative abundance (less than 100 of total sequences). Eleven bacteria genera, including *Pectobacterium*, *Bradyrhizobium*, *Phyllobacterium*, *Ralstonia*, *Sphingomonas*, *Acinetobacter indicus*, *Stenotrophomonas*, *Brevundimonas*, *Achromobacter*, *Staphylococcus*, and *Bacillus* were found exclusively in the starved bed bugs samples (Table 2).

**Table 2:** Taxonomic classification of bacterial sequences (OTUs) that shared in all three samples either from blood fed or starved bed bugs.

Taxonomy (Phylum; class; family; genus)	Number of sequences					
	Blood fed			Starved		
	BB1	BB2	BB3	BB1	BB2	BB3
Proteobacteria; Alpha-proteobacteria; Anaplasmataceae; *Wolbachia*	19,988	29,877	20,038	82,041	82,848	86,363
Proteobacteria; Gamma-proteobacteria; Enterobacteriacese; *Dickeya chrysanthemi*	10,733	12,555	10,791	20,496	18,216	18,317
Proteobacteria; Gamma-proteobacteria; Pseudomonadaceae; *Pseudomonas*	1389	596	808	4000	3164	2907
Proteobacteria; Gamma-proteobacteria; Enterobacteriaceae; *Shimwellia*	95	1	4	–	–	–
Proteobacteria; Gamma-proteobacteria; Moraxellaceae; *Enhydrobacter*	54	2	24	8	4	13
Proteobacteria; Alpha-proteobacteria; Azospirillaceae *Skermanella*	47	2	1	–	–	–
Proteobacteria; Alpha-proteobacteria; Rhodobacteraceae; *Paracoccus*	25	3	2	4	2	18
Proteobacteria; Alpha-proteobacteria; Beijerinckiaceae; *Methylobacterium*	8	3	4	69	55	24
Proteobacteria; Gamma-proteobacteria; Burkholderiaceae; *Curvibacter*	1	1	10	39	64	25
Proteobacteria; Gamma-proteobacteria; Burkholderiaceae; *Pelomonas*	2	1	3	–	–	–
Proteobacteria; Alpha-proteobacteria; Rhizobiaceae; *Mesorhizobium*	3	2	1	–	–	–
Proteobacteria; Gamma-proteobacteria; Enterobacteriaceae; *Pectobacterium*	–	–	–	46	534	92
Proteobacteria; Alpha-proteobacteria; Xanthobacteraceae; *Bradyrhizobium*	–	–	–	9	358	9
Proteobacteria; Alpha-proteobacteria; Rhizobiaceae; *Phyllobacterium*	–	–	–	6	248	4
Proteobacteria; Gamma-proteobacteria; Burkholderiaceae; *Ralstonia*	–	–	–	12	154	1
Proteobacteria; Alpha-proteobacteria; Sphingomonadaceae; *Sphingomonas*	–	–	–	14	94	12
Proteobacteria; Gamma-proteobacteria; Moraxellaceae; *Acinetobacter indicus*	–	–	–	17	13	7
Proteobacteria; Gamma-proteobacteria; Xanthomonadaceae; *Stenotrophomonas*	–	–	–	10	7	1
Proteobacteria; Alpha-proteobacteria; Caulobacteraceae; *Brevundimonas*	–	–	–	6	10	1
Proteobacteria; Gamma-proteobacteria; Burkholderiaceae; *Achromobacter*	–	–	–	1	3	4
Firmicutes; Bacilli; Staphylococcaceae; *Staphylococcus*	–	–	–	12	13	7
Firmicutes; Bacilli; Bacillaceae; *Bacillus*	–	–	–	8	1	16

“–” indicated not found.

**Figure 6:**
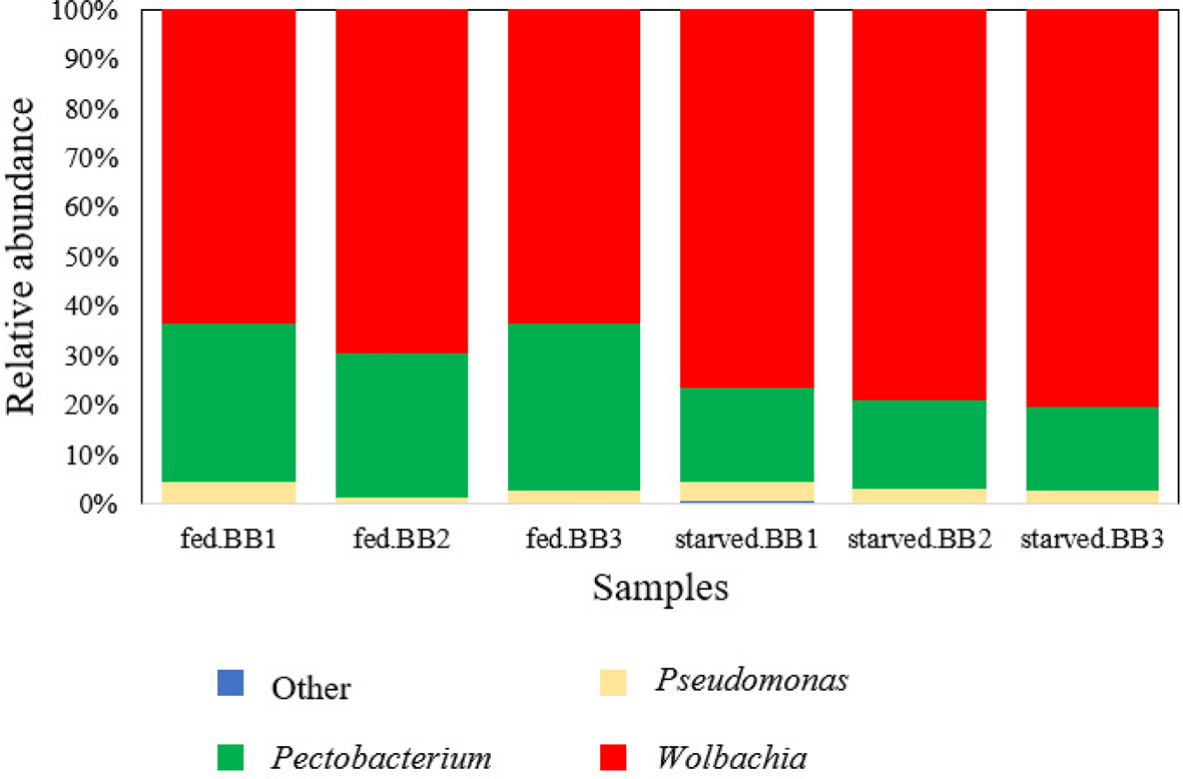
The relative abundance of bacteria genera in each sample from blood-fed bed bugs (start from left, fed.BB1–fed.BB3) and starved bed bugs (starved.BB1–starved.BB3).

The genera, *Wolbachia*, and *Dickeya chrysanthemi* were tremendously dominated the sequence data, with *Wolbachia* containing 19,988, 29,877, and 20,038 sequences and the other, *D. chrysanthemi* containing 10,733, 12,555, and 10,791 sequences for three individuals of blood-fed bed bugs. The number of sequences of these two OTUs obtained from the three starved bed bugs was even higher than the number of sequences acquired from blood-fed bed bugs with 82,041, 82,848, and 86,363 sequences *Wolbachia* and 20,496, 18,216, 18,317 sequences for *D. chrysanthemi* (Table 2). The cumulative sequences from these two OTUs constitute approximately 97% and 96% of all sequences in blood-fed and starved bed bugs (Fig. 6).

Paired sample T-test was performed to determine the significant differences in the number of sequences of bacterial genera present in both blood-fed and starved bed bugs (Table 3). The results showed that the number of *Wolbachia* sequences in starved bed bugs was significantly higher than in blood-fed bed bugs (*p* = 0.004). *Dickeya chrysanthemi* (*p* = 0.023) and *Pseudomonas* (*p* = 0.005) in starved bed bugs were also significantly higher than in blood-fed bed bugs. The number of sequences of *Enhydrobacter* (*p* = 0.329), *Paracoccus* (*p* = 0.869), *Methylobacterium* (*p* = 0.071), and *Curvibacter* (*p* = 0.108) showed no significant differences between blood-fed and starved bed bugs (Table 3).

**Table 3:** The number of sequences between blood-fed and starved bed bugs with *P*-value determined using paired samples T-test.

Genera	Number of sequences (Mean ± SD)		*P*-value
	Blood-fed	Starved	
*Wolbachia*	23,301.00 ± 5695.04	83,750.67 ± 2298.05	0.004
*Dickeya chrysanthemi*	11,359.67 ± 1035.60	19,009.67 ± 1288.19	0.023
*Pseudomonas*	931.00 ± 410.56	3357.00 ± 571.49	0.005
*Enhydrobacter*	26.67 ± 26.10	8.33 ± 4.51	0.329
*Paracoccus*	10.00 ± 13.00	8.00 ± 8.72	0.869
*Methylobacterium*	5.00 ± 2.65	49.33 ± 23.03	0.071
*Curvibacter*	4.00 ± 5.20	42.67 ± 19.76	0.108

Although the number of sequences of dominant genera in starved bed bugs is significantly higher than blood-fed bed bugs, the abundance ratio of the top three most abundant genera of which, *Wolbachia*, *D. chrysanthemi* followed by *Pseudomonas*, accounted for more than 99% of the total sequences between blood-fed and starved bed bugs were similar (Fig. 6).

## Discussion

This study is the first study that characterizes the bacterial community found in blood-fed and starved tropical bed bugs, *Cimex hemipterus*, using high-throughput sequencing. Based on the bacterial community analysis, Proteobacteria, which mainly documented to display antiparasitic activity in insects' guts, was the phylum that predominates in both blood-fed and starved bed bugs^29^.

Despite inter-individual variability (Fig. 3), the variability between individuals is less than 0.01% abundance with no significant difference, and most of the OTUs contain only averaged one to three sequences (data not shown). Since all bed bug samples were fed on the same blood source, taxa variability may occur due to individual variation^30^.

The alpha-diversity in both blood-fed and starved tropical bed bugs was relatively low (Fig. 1)^30,31^. The low bacterial diversity observed may due to the samples used were laboratory strains. Boissière et al. (2012) also reported that bacterial richness and diversity are low in laboratory mosquitoes^30^. Other reasons, such as selective pressures, including microbial interactions or insect immune systems, may also affect the bacterial community's composition^30^.

Beta-diversity estimated based on Bray–Curtis, Weighted- and Unweighted-UniFrac distance matrices showed no significant differences between blood-fed and starved bed bugs when compared using the Mantel test. No significant differences may be due to the dominant genera (more than 99%), of which *Wolbachia*, *D. chrysanthemi*, and *Pseudomonas* were the same between both blood-fed and starved bed bugs. The result suggests that blood-feeding has a limited influence on the bacterial community's overall diversity within tropical bed bugs. Zolnik et al. (2016) and Swei and Kwan (2017) reported similar findings in ticks of which blood-feeding induce limited changes in microbial communities' composition or even reduced the microbiome diversity^32,33^. Although there were no significant differences in alpha and beta-diversity measures between blood-fed and starved bed bugs, the number of bacterial species (the number of OTUs) was lower in blood-fed bed bugs than starved bed bugs (Table 1). The result is also similar to the study by Sontowski and Van Dam (2020) showed a blood meal in *A. aegypti* reduces the diversity and number of OTUs^34^.

There are several bacterial taxa present in both blood-fed and starved bed bugs. The bacterial taxa shared across all samples suggested they probably vertically transmitted, and their presence is probably essential to their host by providing supplementation and maintenance of insects' homeostasis and immune system^35,36^. Among the shared bacterial taxa of blood-fed and starved bed bugs, two OTUs of Proteobacteria dominate the bacterial community of *C. hemipterus*, the alpha-proteobacteria, *Wolbachia*, and gamma-proteobacterium, *D. chrysanthemi*. The identification of *Wolbachia* and a gamma-proteobacteria as the predominant genera in *C. hemipterus* was in accordance with the finding of Hypša and Aksoy^37^, Hosokawa et al.^10^, and Meriweather et al.^18^ in *C. lectularius*, which also reported *Wolbachia* and a gamma-proteobacteria were predominant in common bed bugs. However, the gamma-proteobacterium reported from these studies was unclassified.

*Wolbachia* infections are common in the Cimicidae^38^. As an obligate endosymbiont of bed bugs, *Wolbachia* are vertically transmitted, could be found in many cell types^10,37,39–41^, and play an essential role in host reproduction as well as the production of B vitamins, including biotin and riboflavin^10,41,42^. Thus, the high frequency of *Wolbachia* found in *C. hemipterus* is not surprising but provides evidence that the 16S rRNA assessment is mainly congruent with expectations.

*Dickeya chrysanthemi* (Pectobacterium) was the second most bacteria found in *C. hemipterus*. It is a common plant pathogen and could synthesize siderophores, an iron-chelating agent essential for iron acquiring under iron-limited conditions^43,44^. This characteristic implies their importance in bed bugs and may explain the increased relative abundance of this species in starved bed bugs. The presence of both *Wolbachia* and gamma-proteobacteria and their possible functions in common bed bugs, *C. lectularius*, have been reported by Goodman (2016) of which both *Wolbachia* and the gamma-proteobacteria was essential for bed bugs growth^45^. Moreover, the absence of *Dickeya* leads to reductions in egg production in *C. lectularius*, suggesting that removing the gamma-proteobacteria can reduce fertility^18^.

Other than *Wolbachia* and *Dickeya*, genera present in both blood-fed and starved bed bugs, including *Pseudomonas*, *Methylobacterium*, *Paracoccus*, *Enhydrobacter*, and *Curvibacter* (Table 2). *Pseudomonas* is a generalist, which could be found from the different broad environments. It is also a common pollutant degrader^46^ and can produce siderophore^47,48^. Although *Pseudomonas* has been reported as a contaminant in metagenome studies^49^, *Pseudomonas* found in this study is unlikely a contaminant based on loads of *Pseudomonas* in three starved bed bugs was significantly higher than the blood-fed bed bugs (Table 3). If the *Pseudomonas* was the contaminant, the loads from bed bugs under both blood-fed and starved states should be similar. *Pseudomonas* also could be found in many insects, including mosquitoes^34,50^, beetles^51^, and scolitines^52^, and always linked to detoxifying activities in insect's guts. Infections of *Pseudomonas* are beneficial in these cases, but targeted studies are needed to investigate *Pseudomonas*'s function in tropical bed bugs.

*Methylobacterium* is a common plant growth promoter and could be found in the natural environment ubiquitously^53,54^. The presence of *Methylobacterium* in bed bugs may be necessary as this bacterium is also one of the ten most abundant OTUs characterized by *C. lectularius*^18^. *Paracoccus* has been isolated from industrial effluent, Cayenne ticks, and Tsetse fly^29^. There is no information regarding *Enhydrobacter* and *Curvibacter* in blood-feeding insects, and the role of these bacteria in the gut of blood-feeding insects is not explicit. Nevertheless, bacterial groups consistently present and shared among all the bed bug samples suggest it could serve the host's essential functions.

There were exclusive OTUs in blood-fed and starved bed bugs, even in extremely low abundance (less than 1%) in the overall bacterial community. Genera that only detected in blood-fed bed bugs, including *Shimwellia*, *Mesorhizobium*, *Skermanella*, and *Pelomonas*. According to Brzuszkiewicz et al. (2012), *Shimwellia* can synthesize coenzyme B12 or vitamin B12 de novo^55^. *Mesorhizobium* and *Skermanella* could be found from various ecosystems^29,56^, while *Pelomonas* has been isolated from the assassin bug, *Triatoma maculate*^16^. However, their role in blood-feeding insects is not elucidated.

The bacterial community in starved bed bugs is more diverse and abundant than blood-fed bed bugs. Genera *Acinetobacter*, *Phyllobacterium*, *Achromobacter*, *Bradyrhizobium*, *Staphylococcus*, *Stenotrophomonas*, *Ralstonia*, *Bacillus*, *Sphingomonas*, and *Brevundimonas* were only characterized in starved bed bugs (Table 2). The changes in the bacterial community structure of bed bugs in the starved state could be due to host-driven mechanisms. For instance, the host may produce different antimicrobial proteins, less secretion of mucus on the gut lining, pH changes, and reduced intestines' size during the starved period may alter the bacterial community^19^.

Based on Fig. 6, the abundance ratio of *Wolbachia*, *D. chrysanthemi*, and *Pseudomonas* between the two physiological statuses is similar. However, the number of sequences of these dominant species is significantly higher in starved bed bugs than blood-fed bed bugs (Table 3). The change of bacteria's load may be due to the selection pressure, of which in this study, starvation forced onto the beg bugs. Selection pressure could induce changes in loads of bacteria, and the loads are always higher of samples in the critical stage^57^. The result is in line with the study by Rodgers et al. (2017) on the microbiota of *An. coluzzii*, of which mosquitoes restore their gut homeostasis after the blood meals by excreting bacteria with the blood bolus, result in a 98% reduction in bacterial loads^31^. Tchioffo et al. (2016) also showed blood-fed mosquitoes (*Anopheles*) induced a drop in bacterial diversity and abundance at first and increased later after prolonged starvation^58^.

The decrease of the concentration of symbiotic bacteria may due to the digestive processes that various digestive enzymes killed the symbiotic bacteria. After processing the blood meal, the nutrient supply within the ingested blood may increase the symbiotic bacteria concentration^59^. Besides, the increased concentration of symbiotic bacteria during the starved period could strengthen the host's immune system^19,40^.

Nevertheless, there are also study showing the contrasting result. A study by Fisher et al. (2018) on common bed bugs, *Cimex lectularius*, showed the *Wolbachia* titer declined significantly with prolonged starvation at 40 d after feeding^60^. These studies^31,58,60^ showed that bacterial community composition was affected mainly by arthropod species^61^.

## Conclusion

This study is the first metabarcoding analysis of bacterial communities' characterization from blood-fed and starved tropical bed bugs, *C. hemipterus*. Overall, the bacterial community in both blood-fed and starved bed bugs was dominated by Proteobacteria with dominant genera or species, *Wolbachia*, *D. chrysanthemi*, and *Pseudomonas*. The findings also indicate starvation has a limited effect on the bacterial composition of bed bugs but significantly affects the concentrations of dominant bacterial species. Furthermore, less abundance specific bacterial genera were isolated either from blood-fed or starved bed bugs. It provides information regarding the microbial community that may associate with blood meal digestion and provides fundamentals for future research on the bacterial members' functional roles except for *Wolbachia*.
